# GABBR1 has a HERV-W LTR in its regulatory region – a possible implication for schizophrenia

**DOI:** 10.1186/1745-6150-8-5

**Published:** 2013-02-07

**Authors:** Hedi Hegyi

**Affiliations:** 1 CEITEC—Central European Institute of Technology, Masaryk University, CZ-62500, Brno, Czech Republic; 2 Institute of Enzymology, Research Centre for Natural Sciences, Hungarian Academy of Sciences, Budapest, Hungary

**Keywords:** Schizophrenia, Human endogenous retrovirus, HERV-W, long terminal repeat, LTR, GABA, GABBR1, GABA receptor, Enhancer, Silencer

## Abstract

**Abstract:**

Schizophrenia is a complex disease with uncertain aetiology. We suggest GABBR1, GABA receptor B1 implicated in schizophrenia based on a HERV-W LTR in the regulatory region of GABBR1. Our hypothesis is supported by: (i) GABBR1 is in the 6p22 genomic region most often implicated in schizophrenia; (ii) microarray studies found that only presynaptic pathway-related genes, including GABA receptors, have altered expression in schizophrenic patients and (iii) it explains how HERV-W elements, expressed in schizophrenia, play a role in the disease: by altering the expression of GABBR1 via a long terminal repeat that is also a regulatory element to GABBR1.

**Reviewers:**

This paper was reviewed by Sandor Pongor and Martijn Huynen.

## Background

Schizophrenia is a devastating psychiatric disorder with a strong genetic component (with an estimated 80-85% heritability [1]) affecting approximately 1% of the population. Despite intensive research in the past decades there is still no known cause of the disease that would account for the majority of the cases. Several systematic studies have been conducted that implicated various genes in the aetiology of schizophrenia (in Genecards [2], a comprehensive knowledgebase of all human genes, more than 1600 genes are annotated to have some association with schizophrenia), however only a few of them are considered susceptibility genes [1,3]. In a meta-analysis of more than 8000 European-ancestry subjects significant association with schizophrenia was observed in a region of linkage disequilibrium on chromosome 6p22.1, a region that has been commonly implicated in schizophrenia for a long time [4].

A comprehensive microarray expression profiling of the prefrontal cortex pinpointed a group of genes with abnormal expression [5]. Mirnics et al. found that out of 250 gene groups only genes in the presynaptic pathway showed an altered expression [5]. This is in line with the findings of other groups, calling schizophrenia the disease of the synapse [6].

In a different approach, mostly subscribed to the hypothesis of viral origin of schizophrenia, several groups found a marked expression of human endogenous retroviruses, mostly the HERV-W subtype being expressed in patients with recent onset of schizophrenia [7,8]. Endogenous retroviruses, both HERV-W and HERV-H, were also reported in patients of multiple sclerosis and other types of autoimmune diseases [9,10].

Several studies implied the GABA receptors in the aetiology of schizophrenia. Besides the study by Mirnics et al. [5] other groups also found the GABA receptor B1 gene (GABBR1) to be downregulated in schizophrenic patients [3,11,12].

Several papers implicated the 6p22 chromosomal region in schizophrenia, based on genetic studies of familial cases and large cohorts [4,13]. A review outlined a long genomic region to be associated with the disease, encompassing 25 Mb of the 6p genomic region in question [14]. However, several of the common variants implicated in schizophrenia showing a significant association were not associated with any particular genes [4]. This also leads us to the question of genetic changes that are outside of the coding region of protein-coding genes. This is becoming ever more evident as less single nucleotide variants are found to be associated with genetic diseases that affect the protein sequence than found in regulatory regions [15].

However, only sporadic knowledge existed till now regarding the regulatory regions of genes that might affect the expression of genes implicated in genetic diseases. A recent publication has identified ~2.9 million DNase I hypersensitive sites (DHSs), markers of regulatory DNA in the human genome, based on the coexpression patterns of the regions and known promoters of all human protein-coding genes [16]. The authors did not investigate if these regulatory elements are enhancers, silencers, insulators or other types of regulators.

## Findings

Here we analyzed these regulatory regions of the human genome for the presence of the long terminal region (LTR) of the human endogenous virus type W (HERV-W) as this type of HERV was identified in patients with recent-onset schizophrenia [7]. We used the LTR as query as opposed to the full sequence of HERV-W because it is known to have enhancer/promoter/regulatory functions [17].

Using the HERV-W long terminal repeat (LTR) as query against the regulatory sequences identified in the study of Thurman et al. [16] revealed a full-length match in the regulatory region of GABBR1, the GABA receptor B1 gene (Table 1). GABBR1 is located in the 6p22 chromosomal region that has been implicated in schizophrenia for a long time. As HERVs, especially subtype W, are often found to be differentially expressed in the brain of schizophrenic patients [7], this brings up the possibility that they could trigger the downregulation of GABBR1 via its LTR enhancer/regulator.

**Table 1:** **Regulatory regions for schizophrenia-related genes (as annotated by Genecards**[2]**) that have a full or partial match with the LTR of HERV-W**

**Gene list**	**chr**	**ltrstart**	**ltrstop**	**e-value**	**Score**
GABBR1	chr6	29526020	29526766	0.0	1042
MAPK10	chr4	86878290	86877559	0.0	834
MAPK10	chr4	86878341	86877841	1,00E-160	630
DLD	chr7	107977734	107978228	0.0	706
NRCAM	chr7	107977734	107978228	0.0	706
NR3C2	chr4	148920214	148920590	1,00E-115	464
ATM	chr11	107851843	107852136	2,00E-106	430
SLC18A1	chr8	20016820	20017144	5,00E-83	344
SLC18A1	chr8	20017040	20017144	7,00E-12	82
FAAH	chr1	46856820	46856990	2,00E-44	202
CDC25A	chr3	48377262	48377349	1,00E-27	140
MAP4	chr3	48377349	48377262	1,00E-27	140
SHISA5	chr3	48377262	48377349	1,00E-27	140
MAP2	chr2	210171660	210171694	2,00E-05	58
ACTB	chr7	5832898	5832925	0.003	50

The regulatory regions were taken from Thurman et al. [16]. The LTR sequence ranges (ltrstart, ltrstop), evalues and scores were calculated using the Blastn program [18]. A large fraction of the regulatory regions identified by Thurman et al. [16] were associated with more than one gene and most genes were also associated with more than one regulatory region.

A full search with the HERV-W LTR as query identified 12 other genes with partial LTR matches overlapping enhancer/regulatory sequences identified by [16], listed in Table 1. Besides GABBR1, which had the strongest match with the LTR sequence of HERV-W, several other schizophrenia-related genes had a partial match. Of those, NRCAM, the neural cell adhesion molecule gene has a well-established association with schizophrenia. Barbeau et al. found that its embryonic isoform was severely decreased in the brain of schizophrenic patients [19].

Interpretation. Long terminal repeats (LTRs) of endogenous viruses have long been known to act as enhancers/silencers/regulators of gene expression. However, a lack of comprehensive knowledge of regulatory regions associated with their corresponding gene have been lacking till now. As enhancers can be located hundreds of kilobases away from their target gene, a purely computational approach could not possibly identify such regions. The recent study by Thurman et al. [16] made it possible for the first time to analyze the regulatory regions by querying their sequences with functional elements with known (or suspected) pathogenic functionality.

What is the significance of the HERV-W sequences present in the regulatory regions of genes implicated in schizophrenia? Our hypothesis is that their overexpression at the onset of the disease (triggered by an external factor such as an exogenous virus or stress, etc.) leads to their silencing (or downregulation) by hypermethylation. Transposable element silencing as a consequence of DNA methylation is a well-known phenomenon in mammalian species as it is considered the defense system of the host against its “intragenomic parasites”, as Yoder et al. calls these elements [20]. In this model silencing genes with a HERV in their regulatory region might be the *collateral damage*, so to speak, brought upon by the host in its effort to repress the transcription of the HERV-W endogenous retroviruses, ultimately leading to schizophrenia.

Our finding of a full-length HERV-W LTR in the regulatory region of the GABA receptor B1 gene GABBR1 implicates it in a subset of cases of schizophrenia. This is in accord with several lines of experimental evidence, notably:

(i) The gene is in the 6p22 genomic region that has been implicated most often in schizophrenia in linkage disequlibrium studies [4,13].

(ii) Postmortem studies suggested that schizophrenia is associated with deficits of GABA-mediated synaptic transmission [21].

(iii) GABBR1 was found to be downregulated in several postmortem studies of schizophrenic patients [5,12] and also in autistic brains [22].

Furthermore, our hypothesis of HERV-containing genes silenced by hypermethylation is also supported by experimental evidence, such as:

(iv)  HERV-W RNAs tend to be expressed in the cerebrospinal fluid of recent-onset schizophrenic but not in chronic patients [7].

(v) Besides the decreased expression of GABBR1 and that of the neuron adhesion molecule gene (NRCAM), three other genes in Table 1 with the highest scores were also found to be downregulated: the dihydrolipoamide dehydrogenase (DLD) [23] and the mineralocorticoid receptor (NR3C2) [24] in the prefrontal cortex of schizophrenic patients, whereas the mitogen-activated protein kinase 10 (MAPK10) was found to be silenced in several types of cancer [25].

The significance of evidence (iv) is that the lack of HERVs in chronic patients [7] might be due to silencing by hypermethylation, activated by the overexpression of HERV-W RNA at the onset of the disease. It is interesting to note here that Karlsson et al. found recently an inverse correlation between the levels of transcription of HERV-W LTR intergenic elements and their downstream genes [9].

Our model is also supported by the recently observed overexpression of DNA-methyltransferase 1 (DNMT1) in GABAergic interneurons of schizophrenic brains, apparently actively hypermethylating genes with roles in schizophrenia [26]. Interestingly, one of the genes hypermethylated by DNMT1 is reelin [27], which also contains a HERV-W-like sequence (although not an LTR) in its regulatory region (data not shown).

## Conclusion

Taken together, we provided a new hypothesis for the cause of schizophrenia, surmising that the downregulation of several genes containing a HERV-W LTR in their regulatory region might be brought upon by the host in its effort to suppress the expression of the HERV-W sequences, observed usually only at the onset of the disease. It must be noted that our model can be applied to other types of HERVs and other diseases as well. Notably, we also found that one of the genes in our list, MAPK10 is silenced in several types of cancer. Further studies of gene expression and genome-wide methylation might provide a confirmation (or refutal) of our hypothesis.

## Abbreviations

HERV: Human Endogenous Retrovirus; LTR: Long Terminal Repeat; DHS: DNase I hypersensitive site; GABA: Gamma-Aminobutyric Acid.

## Competing interests

The author declares that she has no competing interests.

## Reviewers’ comments

Review by Sandor Pongor

The author presents a new hypothesis about the role a GABA receptor, GABBR1 might play in the aetiology of schizophrenia. She bases her theory on a new finding that identifies a HERV-W long terminal repeat in the regulatory region of GABBR1. This is an interesting new theory that would help explain how the ubiquitously present HERV-W elements in recent-onset schizophrenics might contribute to the disease via a full-length LTR regulating the expression of a GABA receptor gene. While, if true, the full picture is probably more complex than stated here (the author herself cites the staggering number of more than 1500 genes being involved in schizophrenia), it is an intriguing theory that might be worthwhile to pursue further, in the light of the fact that the aetiology of the disease is still not known. The author may want to include a few ideas regarding how the hypothesis can be tested.

Quality of written English: Acceptable

I thank Prof. Pongor for his favorable review and suggestions. I somewhat toned down the language of the paper and geared it towards a multi-genic model. I included a sentence at the end regarding experiments to test my hypothesis. It is not easy, of course, as animal models are not very accurate, but the recent advances in genome-wide methylation studies could provide an answer.

Review by Martijn Huynen

The manuscript by Hedi Hegyi argues that it explains the how HERV-W elements play a role in Schizophrenia, based on a combination of observations from the literature about links between Schizophrenia, the expression of HERV-W retroviruses after the onset of Schizophrenia, differential expression of GABA receptors in Schizophrenia, the genomic region that has classically been associated with Schizophrenia (6p22.1) and the author’s own analysis of the genome and the distribution of published DNAse I accessible sites over HERV-W LTRs in Schizophrenia associated genes.

My main criticism is that most of the observations that the hypothesis is based on are themselves hypothesis or associations rather than established causal mechanisms. This makes choosing which evidence is relevant and which is not hard to evaluate. In the genomics area there are so many data sets available that one can be rather eclectic in deciding which ones to choose. I am not accusing the author of doing that, but it is not clear to me why which datasets were chosen. I think it is e.g. pointless to take as input a list of 1600 genes potentially related to schizophrenia.

I thank Martijn Huynen for his thorough review and critical comments, I am going to address them one by one.

I took out the considerations about the 1600 genes and the number of protein binding partners as not very relevant to the main findings.

To my understanding, no causal relationships have been established between GABA receptor expression and schizophrenia or between HERV-W retroviruses and schizophrenia, or between any gene in 6p22.1 and schizophrenia.

No causal relationship has been established between schizophrenia and anything yet. That is the point of my paper, to try to establish a causal relationship between the HERVs and schizophrenia. I hope I made my case stronger in this revised version.

At least one of these relationships is not even consistent: one study shows an increased number of GABA receptors in the brain of schizophrenic patients, while another shows a downregulation of the expression of the gene.

Good point. Digging up the evidence about the increased number of GABA receptors made it clear that it was about the GABA A receptors, therefore I removed it as immaterial to my arguments.

The author’s hypothesis, compelling as it may be, therewith appears thus to be build on quicksand. It is not the first time that an analysis of genomics data is based on quicksand, but when so many datasets are combined one would like to see some solid mechanisms. Regarding mechanism: as I understand from the literature, expression of HERV-W and other HERV elements after the onset of Schizophrenia has been repeatedly observed. A direct role of human endogeneous retroviruses in Schizophrenia has not been established, so it is not clear to me which role needs to be explained.

It has not been established because nobody gave a satisfactory explanation yet. That does not mean there is no causal relationship!

All in all I do find the conclusions of the manuscript not very tangible and convincing. Lemma V of the discussion contains three “mights”. To me that is two too many for a convincing hypothesis.

I took out Lemma V as it contained the uncertainty about the increase (GABA A) as opposed to the decrease (GABA B) in the receptors as I explained above. I think (and I hope you agree) my paper and arguments are much more coherent in this revised version, as I was rethinking them and the paper evolved considerably because of that.

Question:

The Thurman study measured DNAse I accessibility in 125 diverse cell and tissue types. Which of these were chosen for the analysis in Table 1?

The Thurman study did not provide information about tissue types for this particular dataset.

Editorial:

Why was only the HERV-W LTR chosen and not the whole genome, because of their role in regulating gene expression? Could the author mention this earlier in the manuscript and provide a solid reference?

Yes. I inserted now an appropriate reference.

synapse, not “synapsis”.

Corrected.

Quality of written English: Needs some language corrections before being published.
